# Oral β-D-glucan potentiates systemic immune responses to intramuscular foot-and-mouth disease vaccination

**DOI:** 10.3389/fvets.2025.1701909

**Published:** 2026-01-08

**Authors:** Hyeong Won Kim, So Hui Park, Mi-Kyeong Ko, Seokwon Shin, Jong-Hyeon Park, Min Ja Lee

**Affiliations:** Center for Foot-and-Mouth Disease Vaccine Research, Animal and Plant Quarantine Agency, Gimcheon-si, Republic of Korea

**Keywords:** foot-and-mouth disease, oral administration, systemic immunity, vaccine, β-D-glucan

## Abstract

**Background:**

Foot-and-mouth disease (FMD) is a viral disease primarily affecting livestock. Although vaccination is the main strategy to control FMD, current commercial FMD vaccines have major drawbacks, such as low antibody titers and short antibody titer maintenance periods. We hypothesized that these shortcomings were caused by low systemic immune induction and the absence of mucosal immune induction by the FMD vaccine.

**Methods:**

To address the limitations, we enhanced adaptive immune responses through the oral administration of β-D-glucan (BDG). We evaluated the systemic and mucosal immune response of the BDG-fed group (BDG intake + FMD vaccine) and control groups (FMD vaccine) *in vivo* and *ex vivo* using mice and pigs.

**Results:**

The results showed that the oral administration of BDG induced long-term antibody and virus-neutralizing antibody titers in mice and pigs through robust cellular and humoral immunity. We showed that the BDG intake stimulates secretory IgA production in mice and pigs. We also showed that mucosal and systemic immunity-related genes were upregulated by the BDG intake.

**Conclusion:**

In this study, we provide evidence that the oral administration of BDG improves the overall efficacy of the FMD vaccine by inducing mucosal immunity, which enhances systemic immunity, leading to robust cellular and humoral immune responses in the host. This study is relevant to the establishment of new FMD vaccination strategies, programs, and policies and will contribute to improving field challenges.

## 1 Introduction

Foot-and-mouth disease (FMD) is a highly contagious disease caused by the FMD virus (FMDV) (1). FMD mainly affects artiodactyl livestock and wildlife, and its clinical symptoms include blistering, fever, and loss of appetite (2). FMDV is classified into seven serotypes: O, A, C, Asia1, SAT1, SAT2, and SAT3. FMDV replicates rapidly in the host after infection, causing the clinical symptoms of FMD (3, 4). Among the seven FMDV serotypes, O and A serotypes occur mainly in Korea (5). Countries where FMD is prevalent, including Korea, adopted vaccination as a strategy to control FMD. However, most commercially available FMD vaccines currently distributed in farms are parenteral vaccines. Oral vaccine delivery eliminates the need for specialized personnel to administer the vaccine and prevents infections caused by contaminated needles and syringes (6). Oral vaccines directly stimulate the mucosal surface to elicit a strong mucosal immune response. Mucosal immunity is particularly important because most viruses (e.g., FMDV) invade mucosal surfaces (7). As the mucosa is the first line of defense of the host against viral infection, eliciting robust cellular and humoral immune responses at these mucosal sites is critical for blocking pathogen invasion via mucosal routes (8–10).

To develop a powerful and effective vaccine, the selection of an adjuvant is of the utmost importance. An adjuvant is included in the vaccine composition to improve the immune response induced by an antigen and to extend the duration of immunity of the vaccine. The selection of a suitable adjuvant has the advantage of reducing the frequency of booster vaccinations and the number of antigens included in the vaccine (11, 12). In an earlier study, we found that an FMD vaccine containing BDG as an adjuvant induced robust innate and adaptive immunity. We also confirmed that the FMD vaccine containing BDG promotes the secretion of antigen-specific IgA (13).

Dendritic cell-associated C-type lectin-1 (Dectin-1) is a receptor that specifically recognizes polysaccharides, including BDG, and is expressed on myeloid [macrophages, neutrophils, dendritic cells (DCs)] and lymphoid (γδ T cells) lineage cells (14–17). Ligand-stimulated Dectin-1 activates the expression and secretion of many cytokines (IL-1β, IL-2, IL-10, and IL-12) involved in innate and adaptive immunity through downstream signaling (18–20). BDG is a naturally occurring polysaccharide resistant to enzymatic degradation (21). BDG is readily internalized by the host immune cells (22). As a natural product, BDG has the advantages such as high biocompatibility, few side effects, and high affinity for immune cells (23). Therefore, BDG was selected as an oral immunostimulant.

The FMD vaccine used in the experiment was manufactured using the same formula as the commercial FMD vaccine. A majority of commercial FMD vaccines are inactivated vaccines using chemically inactivated whole FMDV as an antigen. In addition to inactivated antigens, FMD vaccines contain ISA206, aluminum hydroxide (AL), and Quil-A to maintain antigen stability, slow antigen release, and improve vaccine efficacy. ISA206, a mineral oil-based oil adjuvant, induces a stronger immune response than that of conventional oil adjuvants. However, ISA206 not only promotes the degradation of inactivated antigen particles (146S) but also causes serious side effects such as edema and necrosis at the vaccination site when used in large amounts (24, 25). AL adsorbs antigens, improves antigen stability, and induces Th2-mediated immunity. However, AL can cause neurotoxicity due to an excessive inflammatory response at the vaccination site. Quil-A, which is produced by purifying crude saponins, has low toxicity and induces a strong immune response (26–29). Therefore, commercial FMD vaccines have limitations, such as a lack of Th1-mediated immunity and side effects due to the adjuvants contained in the vaccine.

In this study, the side effects of the FMD vaccine were improved by reducing the dose of adjuvants contained in the vaccine by setting a base dose of half the recommended dose of the FMD vaccine. We also improved the depressed immune response caused by low-dose FMD vaccination by the oral administration of BDG. The immune response induced by the BDG intake was observed by measuring antibody (Ab) and virus-neutralizing (VN) titers in mice and pigs. The safety of the hosts was confirmed through body weight (BW), food intake, and biochemical tests (the liver and kidney function tests). We also evaluated secretory IgA (SIgA) concentrations in mice and pigs to determine the mucosal immunity elicited by the BDG intake and further evaluated the expression levels of mucosal immunity-related genes in peripheral blood mononuclear cells (PBMCs) from pigs. This study aimed to evaluate whether oral BDG enhances immune responses and the protective efficacy of an inactivated FMD vaccine in pigs and mice.

## 2 Materials and methods

### 2.1 Animals

C57BL/6 N female mice were purchased from KOSA BIO Inc. (Gyeonggi-do, Republic of Korea). Landrace pigs were purchased from BARON BIO Inc. (Gyeongsangbuk-do, Republic of Korea). The animals were housed in a biosafety level 3 (BSL3) facility at the Animal and Plant Quarantine Agency (13). Saliva was collected after pilocarpine hydrochloride (100 μg/dose; Sigma-Aldrich, MO, USA) was injected intraperitoneally into mice to stimulate saliva secretion.

### 2.2 Cells and viruses

The fetal porcine kidney cell line (LF-BK), fetal goat tongue epithelium (ZZR 127), baby hamster kidney (BHK-21), FMDV serotype O (O PA2; GenBank accession No. AY593829.1), and serotype A (A YC; GenBank Accession No. KY766148.1) were used in this study. FMDV serotype O (O/VET/2013) was used as a mouse challenge virus. Cells and viruses were cultured in Dulbecco’s modified Eagle’s medium (HyClone, UT, USA) (13).

### 2.3 Antigen purification

The BHK-21 cells were infected with FMDV (O PA2 and A YC). Sixteen h after viral inoculation, the cultures were treated twice with 0.003 N binary ethyleneimine (0.003 N) to achieve inactivation. The inactivated viruses were concentrated using polyethylene glycol 6,000 (Sigma-Aldrich). Antigen purification was performed using a 15–45% sucrose density gradient, followed by ultracentrifugation. Each gradient fraction was examined with BioSign FMDV Ag (Princeton BioMeditech, NJ, USA) to verify the presence of inactivated virions. The 146S antigen was quantified by spectrophotometry at 259 nm. Final inactivation was confirmed using ZZR 127 and BHK-21 cells, ensuring that no infectious virus remained in the processed supernatants (13).

### 2.4 Formulation of the test vaccine

The test vaccine used in the mouse study comprised purified 0.375 μg antigens (O PA2 and A YC, respectively), 15 μg Quil-A (InvivoGen, CA, USA), 10% AL, and ISA206 (Seppic, Paris, France; 50% w/w). One dose comprised a total volume of 100 μL. The test vaccine used in the pig study comprised purified 15 μg antigens (O PA2 and A YC, respectively), 150 μg/dose Quil-A, 10% AL, and ISA206 (50% w/w). One dose comprised a total volume of 1 mL (13).

### 2.5 Food efficiency ratio in mice and the liver and kidney function tests in pigs

During the mouse study, food intake was measured daily, and weight gain was recorded weekly until 28 days post-vaccination (dpv) and monthly until 56 dpv (30). The food efficiency ratio (FER) was calculated using the following equation:


$$\mathrm{FER}=\frac{\text{Weight gain}\;(\text{grams})}{\text{Food intake}\;(\text{grams})}\times 100$$

To confirm the safety of BDG intake, we performed the liver and kidney function tests at KLS BIO, Inc. (Gyeonggi-do, Republic of Korea). Serum albumin (ALB), blood urea nitrogen (BUN), alanine aminotransferase (ALT), aspartate aminotransferase (AST), albumin/globulin (A/G), total protein (TP), lactate dehydrogenase (LDH), and creatinine (CREA) levels were evaluated with a HITACHI Automatic Analyzer 3,100 (Hitachi High-Tech Corporation, Tokyo, Japan).

### 2.6 PBMC isolation

Blood samples were collected from pigs and processed according to established protocols (13). PBMCs were isolated using Ficoll-Paque^PLUS^ (Cytiva, MA, USA). Ammonium–chloride–potassium lysis buffer was used to eliminate red blood cells (Gibco™; Thermo Fisher Scientific, Inc., MA, USA). Total cells were counted using a Bio-Rad TC20 cell counter (Bio-Rad Laboratories, CA, USA). Isolated cells were dissolved in TRIzol reagent (Invitrogen, CA, USA) and stored at −80 °C.

### 2.7 Serological assays

To assess Ab titers against FMDV antigen (serotype O and A) in serum, PrioCheck™ FMDV kits (Prionics AG, Schlieren, Switzerland) were used as per the instructions. Results were obtained at 450 nm and converted to percent inhibition (PI) values. For the PrioCheck™ kits, a PI value ≥50% was regarded as Ab-positive (13).

The VN assay was performed following the procedures outlined by the World Organization for Animal Health (31). Sera were heat inactivated and then serially diluted. Subsequently, FMDV (1 TCID_50_/50 μL) was added and incubated for 1 h. LF-BK cells (10^4^ cells/50 μL) were seeded and incubated for 3 days. Afterward, cytopathic effects were checked (13, 32). Host defense against FMDV infection is considered achievable when VN titers exceed 1.65 (Log_10_) (33).

### 2.8 Secretory IgA concentration assays

To assess the concentrations of murine and porcine SIgA in the sera, an SIgA ELISA kit (Cusabio, Wuhan, China) was used as per the instructions. The concentration of SIgA in mouse saliva was assessed using an SIgA ELISA kit (Cloud-Clone Corp., TX, USA), as per the user manual. Results were obtained at 450 nm using a spectrophotometer (Hidex, Turku, Finland) (34, 35).

### 2.9 Long-term immunity assessment and FMDV challenge in mice

Mice in both the vaccine-only group (positive control) and the BDG-fed group received the test vaccine via the intramuscular (IM) route. The negative control group received an equal volume of PBS. In the BDG-fed group, oral administration of BDG (Sigma-Aldrich; 100 μg/dose) was performed by pipetting daily until 28 dpv and at weekly intervals from 28 to 56 dpv. Blood samples were collected at 0, 7, 14, 21, 28, 35, 42, 56, 70, and 84 dpv for serological analysis (13).

To assess long-term host defense against viral infection, FMDV challenges were performed as per a previously described protocol (13). All groups of mice were challenged with FMDV [100 lethal dose 50% (LD_50_) O/VET/2013] via the intraperitoneal route at 84 dpv. BWs and survival rates were observed and recorded for up to 7 days post-challenge (dpc).

### 2.10 Long-term immunity assessment in pigs

Pigs in both the vaccine-only group and the BDG-fed group received the test vaccine via the IM route. The negative control group received an equal volume of PBS. In the BDG-fed group, oral administration of BDG (Sigma-Aldrich; 20 mg/dose) was performed using a zonde once daily until 28 dpv and subsequently at one-week intervals until 56 dpv. Following the initial immunization, a booster dose was administered at 28 dpv via the identical route. For serological analysis, blood collection was performed from the vaccinated pigs at 0, 7, 14, 21, 28, 35, 42, 56, 70, and 84 dpv (13).

### 2.11 Quantitative RT-PCR

RNA was purified from TRIzol reagent (Invitrogen) with the RNeasy Mini Kit® (QIAGEN, CA, USA) following the manufacturer’s protocol. cDNA was synthesized using the GoScript Reverse Transcription System (Promega, WI, USA), as per the manufacturer’s protocol. Subsequently, quantitative RT-PCR (qRT-PCR) was conducted with SYBR Green Supermix (Bio-Rad) (13). Gene expression data were normalized to HPRT (reference gene) levels and expressed as fold changes relative to the control group. All primers used in the experiments are detailed in Supplementary Table 1.

### 2.12 Statistical analysis

Except where noted, results are shown as the mean ± SEM. Survival curves were drawn using the Kaplan–Meier method, and differences were analyzed using the log-rank sum test. Results between the groups were analyzed using one-way or two-way analysis of variance and Tukey’s or Dunnett’s *post hoc* tests. Statistical significance is indicated by ^*^p < 0.05, ^**^p < 0.01, ^***^p < 0.001, and ^****^p < 0.0001. Results were calculated using GraphPad Prism 10.2.3 (GraphPad, San Diego, CA, USA).

## 3 Results

### 3.1 Host well-tolerated the BDG intake

To assess the safety effects of BDG intake in mice, food intake and BW were monitored, and the FER was calculated based on the oral administration schedule. The BW of the mice was measured weekly until 28 dpv and monthly until 56 dpv (Supplementary Table 2). Throughout the experiment, the BDG-fed group showed no significant differences in FER compared to those of the control groups (Supplementary Table 3). These results demonstrate that the BDG intake schedule (100 μg/dose) implemented in this study did not cause side effects in mice. To evaluate the side effects of the BDG intake according to the oral administration schedule in pigs, host health status was checked through the liver and kidney function tests. As the oral administration of BDG progressed, the liver and kidney functional indices (ALB, AST, ALT, LDH, TP, A/G ratio, BUN, and CREA) of pigs at each time point (28, 56, and 84 dpv) showed no significant difference in the BDG-fed group compared to that of the other control groups (Table 1). These results demonstrated that the BDG intake is safe for pigs.

**Table 1:** Safety of BDG verified through serum biochemical tests in pigs.

Group	Days post-vaccination (dpv)	ALT (U/L)	AST (U/L)	BUN (mg/dL)	CREA (mg/dL)	LDH (U/L)	TP (mg/dL)	ALB (mg/dL)	A/G ratio
NC	0	41.20 ± 3.68	40.00 ± 2.06	5.90 ± 0.37	0.75 ± 0.02	421.28 ± 45.74	2.58 ± 0.10	2.90 ± 0.06	1.22 ± 0.04
	28	56.80 ± 4.03	46.33 ± 12.25	9.56 ± 1.08	1.01 ± 0.05	496.23 ± 40.97	6.08 ± 0.15	3.36 ± 0.05	1.26 ± 0.08
	56	49.60 ± 1.85	43.20 ± 13.28	10.50 ± 1.21	1.15 ± 0.02	333.10 ± 13.02	6.72 ± 0.15	3.14 ± 0.08	0.89 ± 0.05
	84	47.00 ± 1.74	46.00 ± 14.04	17.26 ± 1.47	1.46 ± 0.05	266.48 ± 2.76	6.06 ± 0.07	3.62 ± 0.04	1.49 ± 0.05
PC	0	37.20 ± 1.73	32.60 ± 0.61	7.50 ± 1.43	0.68 ± 0.02	340.30 ± 13.22	5.34 ± 0.07	2.98 ± 0.07	1.26 ± 0.08
	28	48.40 ± 1.85	45.50 ± 3.32	9.30 ± 0.67	0.93 ± 0.02	456.34 ± 17.62	6.30 ± 0.14	3.32 ± 0.11	1.12 ± 0.07
	56	53.80 ± 3.33	48.60 ± 9.20	13.98 ± 0.95	1.16 ± 0.05	361.90 ± 26.52	6.80 ± 0.10	3.36 ± 0.08	0.98 ± 0.02
	84	43.60 ± 2.79	47.75 ± 5.86	16.78 ± 1.60	1.30 ± 0.07	254.12 ± 8.38	6.34 ± 0.12	3.72 ± 0.09	1.44 ± 0.08
Exp	0	41.00 ± 3.24	42.60 ± 2.44	3.92 ± 0.63	0.80 ± 0.04	348.72 ± 21.02	5.78 ± 0.21	3.02 ± 0.12	1.15 ± 0.13
	28	43.80 ± 4.13	42.00 ± 3.67	6.44 ± 0.58	0.98 ± 0.05	433.60 ± 51.40	6.34 ± 0.18	3.02 ± 0.28	0.98 ± 0.15
	56	56.60 ± 4.07	43.00 ± 6.84	10.88 ± 1.04	1.21 ± 0.05	426.25 ± 23.44	7.14 ± 0.12	3.58 ± 0.09	1.05 ± 0.05
	84	37.20 ± 4.16	43.00 ± 3.36	15.14 ± 1.39	1.41 ± 0.03	238.36 ± 6.28	6.50 ± 0.19	3.80 ± 0.15	1.41 ± 0.05

Pigs were divided into three groups: negative control (NC; PBS), positive control (PC; FMD vaccine only), and experimental (Exp; FMD vaccine + BDG intake) groups. Data are represented as the mean ± SEM of triplicate measurements (*n* = 5–6/group). Statistical analyses were performed using one-way ANOVA, followed by Tukey’s *post hoc* test. Different superscripts represent significant differences at *p* < 0.05.

### 3.2 BDG intake elicits a robust humoral immune response in mice

**Figure 1:**
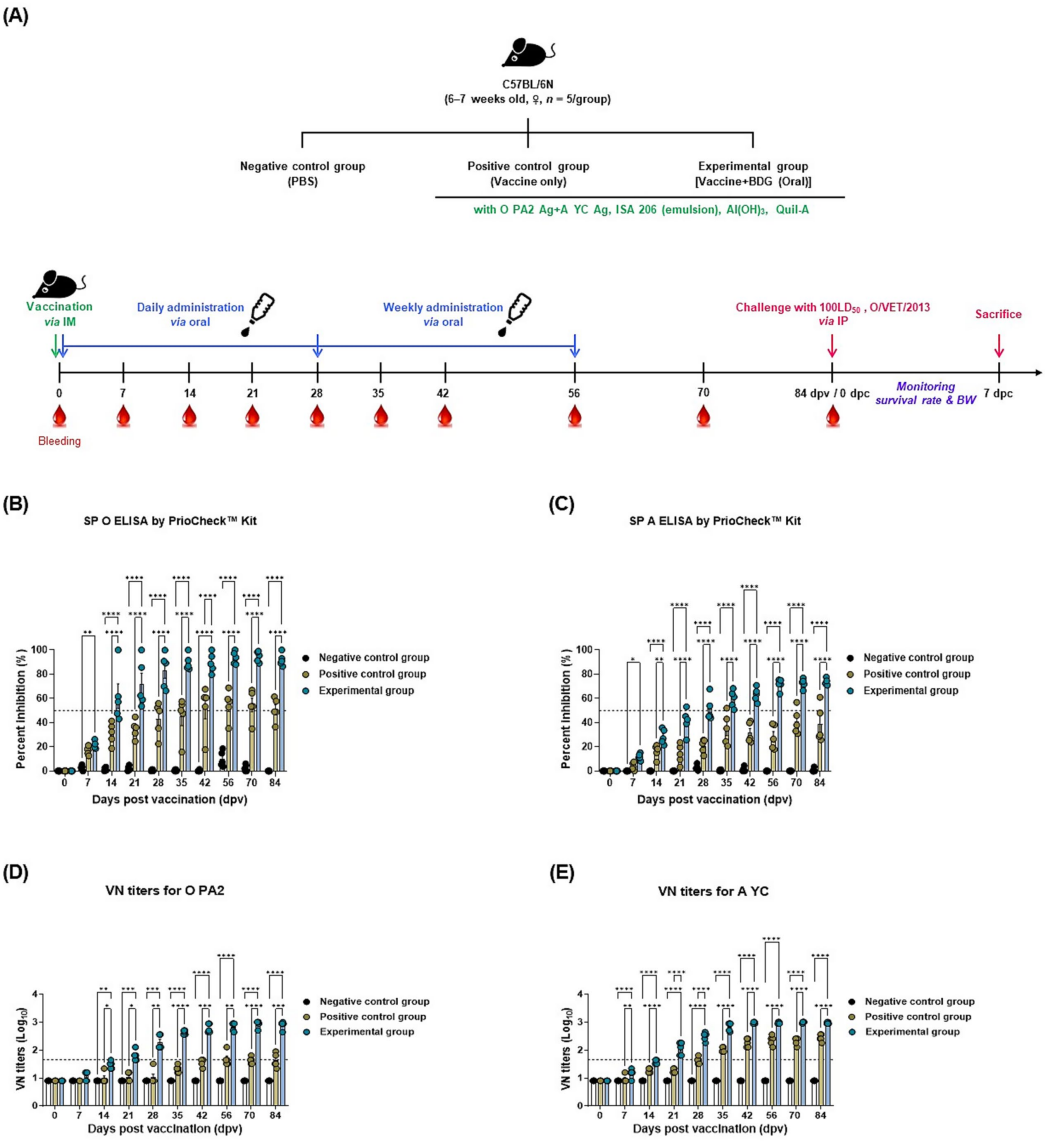
BDG intake enhances the efficacy of the FMD vaccine, eliciting robust long-lasting immunity in mice. Mice were divided into three groups: negative control (NC; PBS), positive control (PC; FMD vaccine only), and experimental (Exp; FMD vaccine + BDG intake) groups. Mice (Exp and PC groups) were vaccinated with the FMD vaccine via the intramuscular route. Blood was collected at 0, 7, 21, 28, 35, 42, 56, 70, and 84 dpv for serological analysis using SP ELISA kits and VN tests. **(A–C)** Experimental strategy **(A)**; antibody titers, as determined using SP O ELISA kits **(B)**; SP A ELISA kits **(C)**; VN titers for O PA2 **(D)**; and VN titers for A YC **(E)**. Data are presented as the mean ± SEM of triplicate measurements (*n* = 5/group). Statistical analyses were performed using two-way ANOVA, followed by Tukey’s *post hoc* test. ^*^p < 0.05; ^**^p < 0.01; ^***^p < 0.001; ^****^p < 0.0001.

To evaluate the humoral immunity induced by the BDG intake, experiments were conducted as per the design shown in Figure 1A and Supplementary Table 4. The BDG-fed group showed a steeper increase in Ab titers and converted to Ab positivity than the vaccine-only group. The BDG-fed group also showed potent adaptive immunity and maintained antibody positivity until 84 dpv (Figures 1B,C). Similar to the Ab titers, VN titers specific for FMDV (O PA2 or A YC) also increased more rapidly in the BDG-fed group and were maintained for a longer period than those in the control groups (Figures 1D,E). These results demonstrate that the BDG intake enhances vaccine-induced immune responses, leading to robust humoral immunity.

### 3.3 BDG intake induces potent host defense at the long-term stage in mice

**Figure 2:**
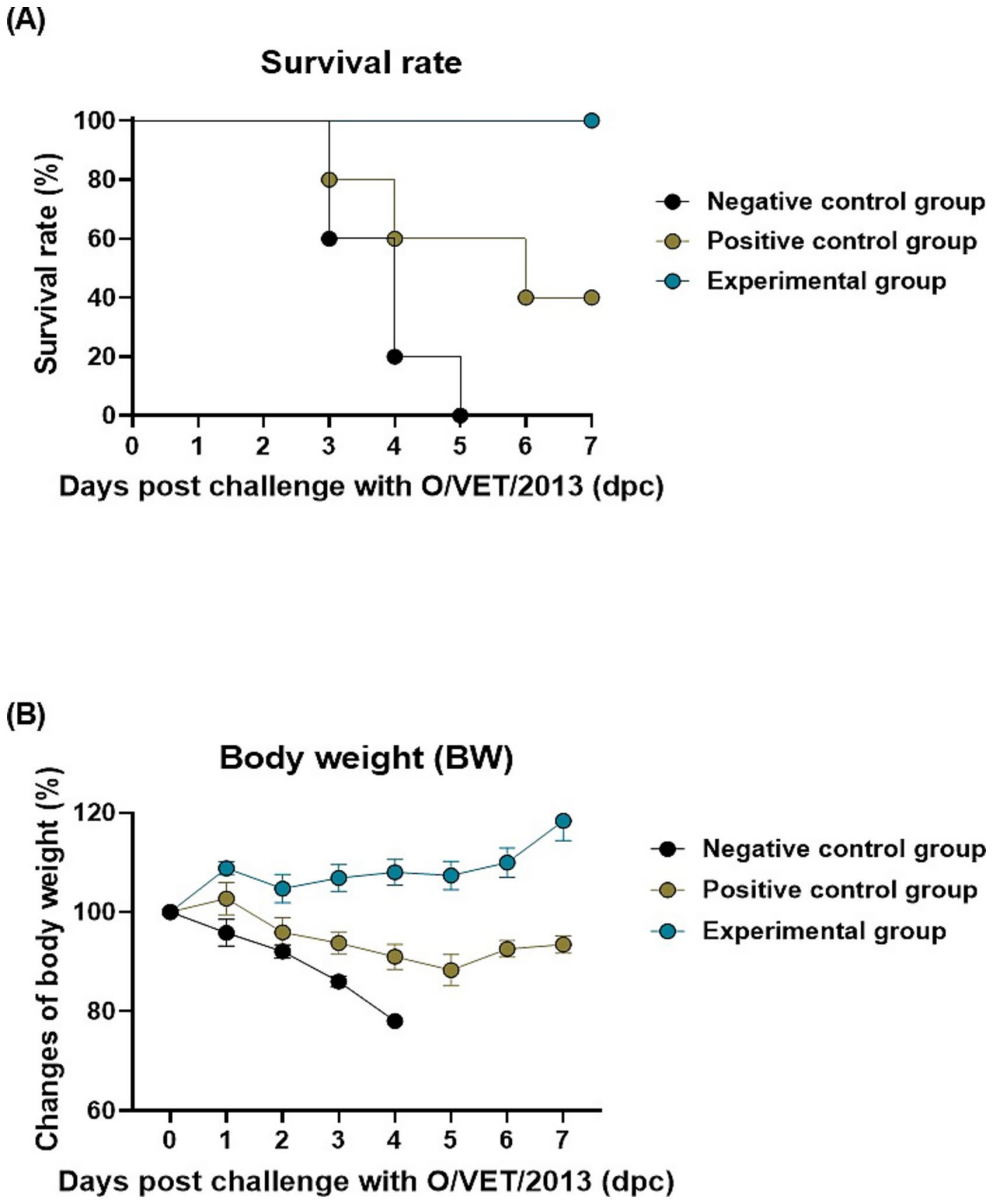
BDG intake enhances the efficacy of the FMD vaccine, eliciting potent host defense in mice. Mice were divided into three groups: negative control (NC; PBS), positive control (PC; FMD vaccine only), and experimental (Exp; FMD vaccine + BDG intake) groups. FMD vaccines were injected via the intramuscular route into mice later challenged with FMDV O (100 lethal dose 50% [LD_50_] O/VET/2013) at 84 dpv via the IP route. The survival rates and body weights were monitored at 7 dpc. **(A–C)** Experimental strategy **(A)**, survival rates post-challenge with O/VET/2013 **(B)**, and changes in body weight post-challenge with O/VET/2013 **(C)**. The data are presented as the mean ± SEM (*n* = 5/group).

To evaluate the long-term host defense elicited by the oral administration of BDG in mice, experiments were conducted as per the strategy shown in Figure 1A. The BDG-fed, vaccine-only, and naive groups exhibited 100, 40, and 0% survival rates, respectively, against FMDV serotype O infection (Figure 2A). In the challenge experiment, no significant difference in BW was observed among the groups (Figure 2B). These results demonstrate that the BDG intake contributes to long-term host defense.

### 3.4 BDG intake elicits robust humoral immunity in pigs

**Figure 3:**
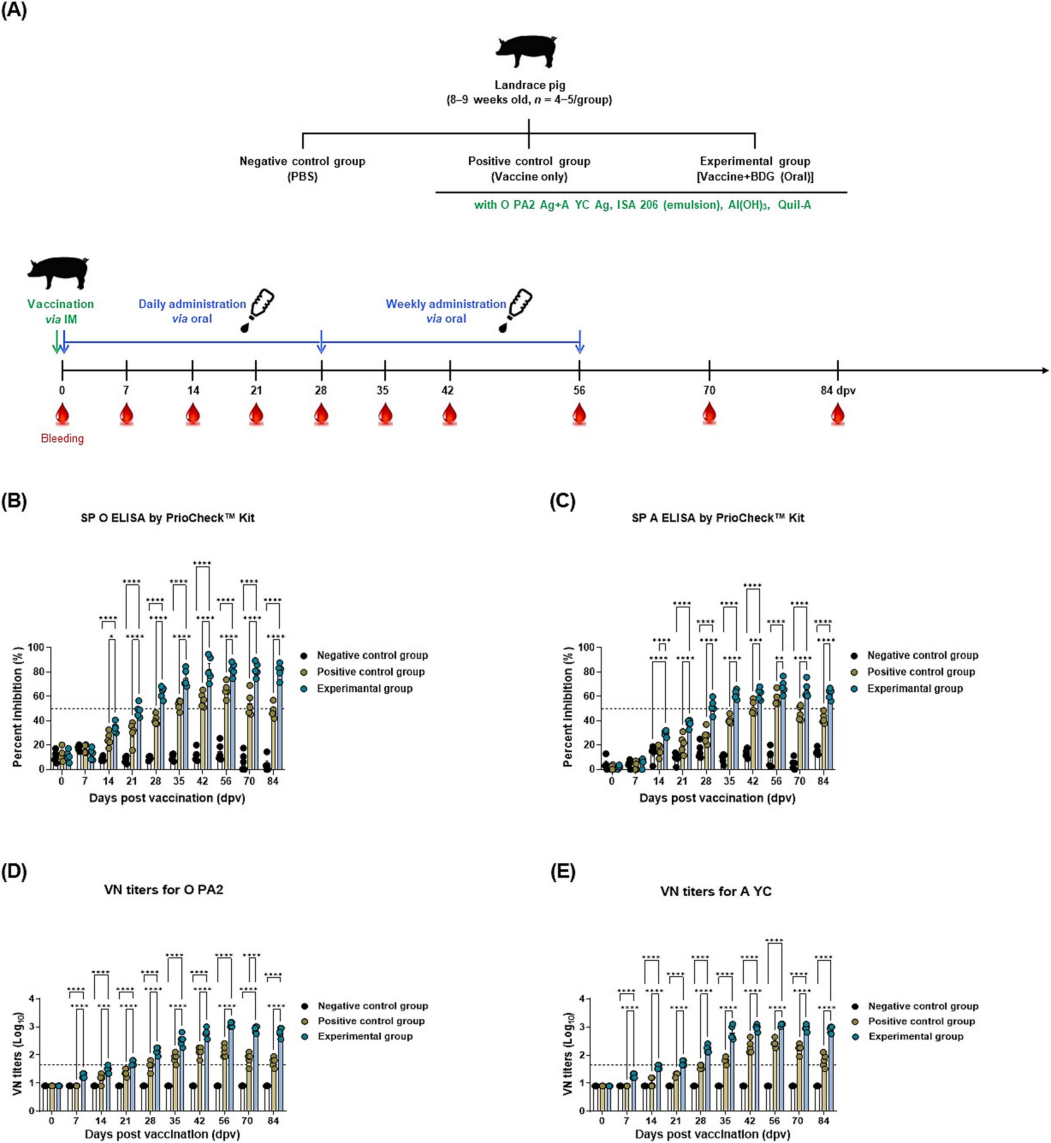
BDG intake enhances the efficacy of the FMD vaccine, eliciting robust long-lasting immunity in pigs. Pigs were divided into three groups: negative control (NC; PBS), positive control (PC; FMD vaccine only), and experimental (Exp; FMD vaccine + BDG intake) groups. Vaccination was performed twice at 28-day intervals, with 1 mL of vaccine (one dose) injected via the deep intramuscular route into the necks of the animals. Blood samples were collected from pigs at 0, 7, 14, 21, 28, 35, 42, 56, 70, and 84 dpv for serological assays. **(A–C)** Experimental strategy: **(A)** antibody titers, as determined using SP O ELISA kits **(B)**; SP A ELISA kits **(C)**; VN titers for O PA2 **(D)**; and VN titers for A YC **(E)**. Data are presented as the mean ± SEM of triplicate measurements (*n* = 5–6/group). Statistical analyses were performed using two-way ANOVA, followed by Tukey’s *post hoc* test. ^*^p < 0.05; ^**^p < 0.01; ^***^p < 0.001; and ^****^p < 0.0001.

To evaluate the humoral immunity induced by the BDG intake, experiments were performed as per the strategy shown in Figure 3A and Supplementary Table 4. BDG-fed and vaccine-only groups showed similar Ab titers, with a gentle increase up to 14 dpv. From 21 dpv (serotype O) and 28 dpv (serotype A) onwards, the BDG-fed group gradually became Ab-positive, whereas the vaccine-only group showed subjects with maintained or even decreasing Ab titers. After the booster immunization, the BDG-fed group showed higher Ab titers than the other groups up to 84 dpv. The BDG-fed group also maintained elevated Ab titers over a long period without a rapid decline. The vaccine-only group also showed an increase in Ab titers after the second vaccination, although the levels were significantly lower than those in the BDG-fed group (Figures 3B,C). For the VN titers specific to FMDV serotype O (O PA2) or serotype A (A YC), the overall VN titer results were similar to those of the Ab titer (Figure 3D). The BDG-fed group showed a steady increase in VN titers from 7 dpv and high VN titers until 84 dpv, whereas the vaccine-only group showed an increase in VN titers from 21 dpv and a gradual decrease from 42 dpv. These results demonstrate that the potent humoral immunity elicited by the oral administration of BDG enhances the systemic immunity induced by FMD vaccination, thereby stimulating adaptive immunity in the host.

### 3.5 BDG intake stimulates the secretion of SIgA in mice and pigs

**Figure 4:**
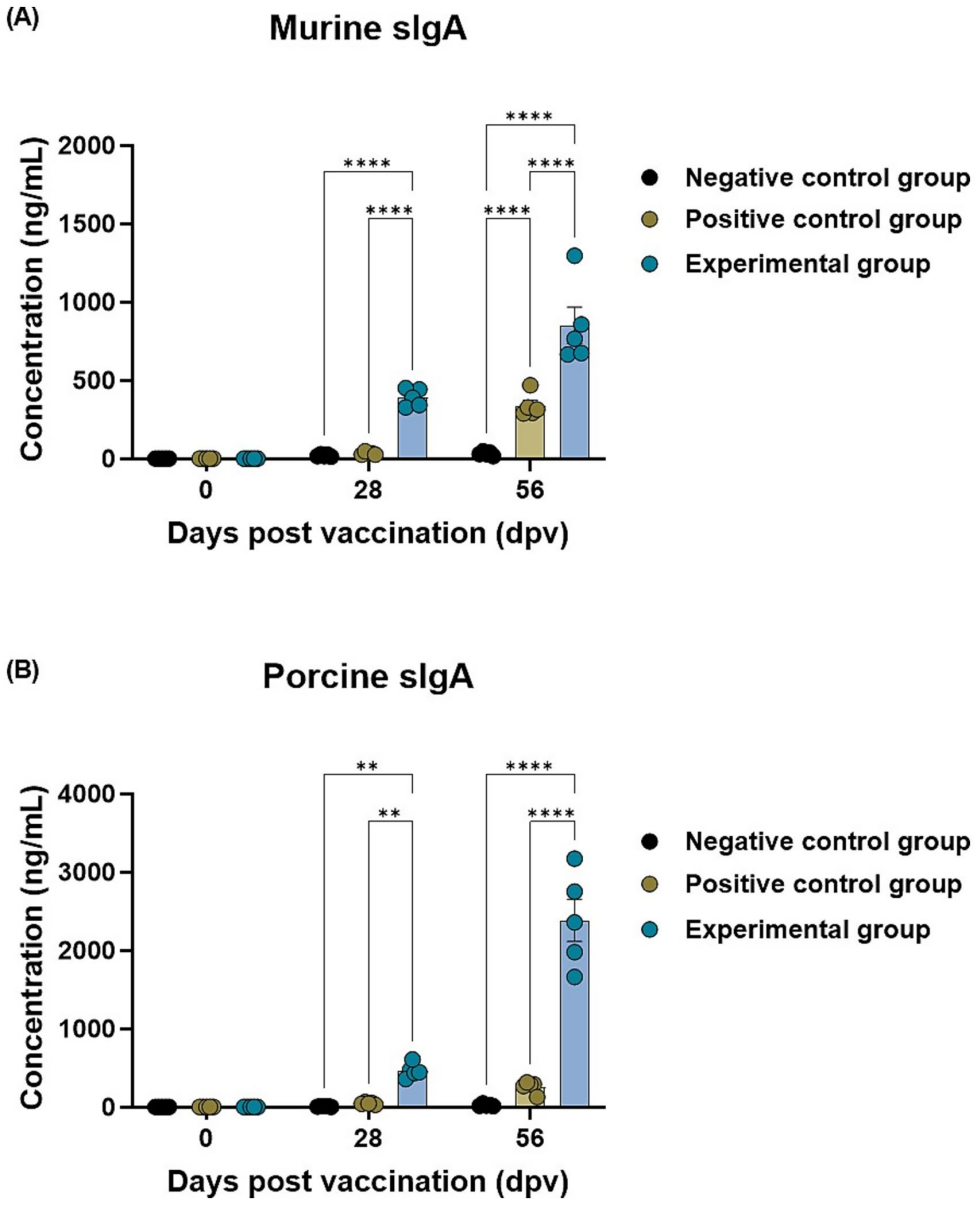
BDG intake enhances the efficacy of the FMD vaccine, eliciting high secretory IgA concentrations in mice and pigs. The experimental strategy and methods are described in the legends of Figures 1A, 3A. **(A,B)** murine secretory IgA **(A)** and porcine secretory IgA **(B)** concentrations. Data are presented as the mean ± SEM of triplicate measurements (*n* = 5–6/group). Statistical analyses were performed using two-way ANOVA, followed by Tukey’s *post hoc* test. ^**^p < 0.01; ^****^p < 0.0001.

To evaluate whether the oral administration of BDG elicits systemic immunity in the host, SIgA secretion was determined in mice and pigs. The SIgA concentrations measured in mice sera showed that the BDG-fed group had significantly higher SIgA concentrations than those in the control groups at both 28 and 56 dpv (Figure 4A). The SIgA concentrations measured in mouse saliva were higher in the BDG-fed group than in the control groups at 56 dpv (Supplementary Figure 1). The SIgA concentrations measured in pigs were also significantly higher in the BDG-fed group than those in the control group at both 28 and 56 dpv (Figure 4B). These results demonstrate that the BDG intake activates systemic immunity in the host, inducing long-term SIgA secretion.

### 3.6 BDG intake stimulates the expression of mucosal and systemic immune-related genes

**Figure 5:**
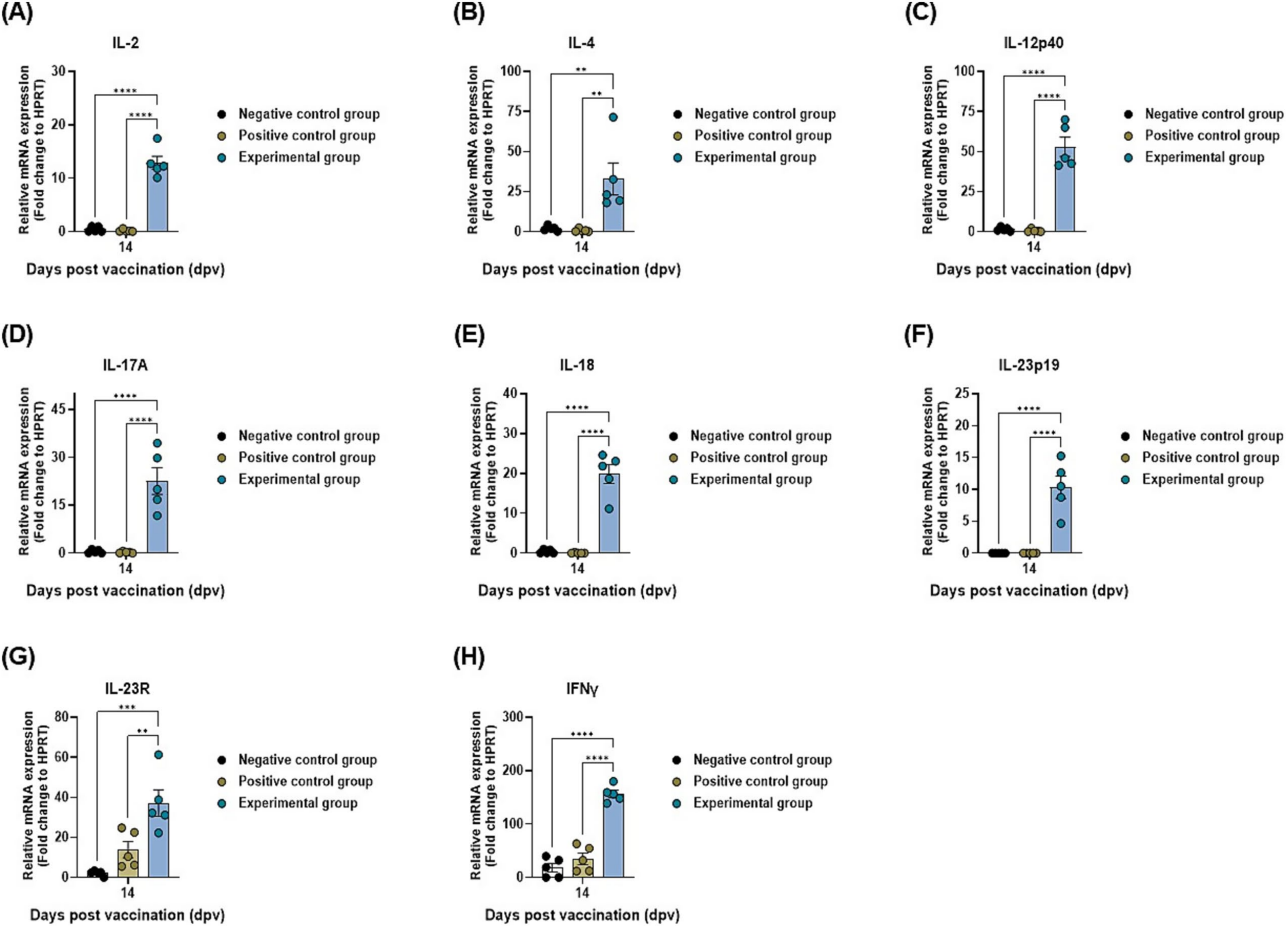
BDG intake enhances the efficacy of the FMD vaccine, upregulating the expression of systemic immune-related genes. Porcine PBMCs isolated from the whole blood of vaccinated pigs (*n* = 5–6/group), as described in Figure 3A, were used for qRT-PCR. Gene expression levels were normalized to those of HPRT and presented as ratios relative to control levels. **(A–H)** Gene expression levels of IL-2 **(A)**; IL-4 **(B)**; IL-12p40 **(C)**; IL-17A **(D)**; IL-18 **(E)**; IL-23p19 **(F)**; IL-23R **(G)**; IFNγ **(H)**. Statistical analyses were performed using two-way ANOVA, followed by Tukey’s *post hoc* test. ^**^p < 0.01; ^***^p < 0.001; and ^****^p < 0.0001.

To identify cytokines related to mucosal and systemic immunity induced by oral administration of BDG, the gene expression levels of cytokines were evaluated using qRT-PCR (Figure 5A−H). Samples were collected at 14 dpv as shown in Figure 3A. The expression of mucosal and systemic immune-related cytokines, such as IL-2 (Figure 5A), IL-4 (Figure 5B), IL-12p40 (Figure 5C), IL-17A (Figure 5D), IL-18 (Figure 5E), IL-23p19 (Figure 5F), IL-23R (Figure 5G), and IFNγ (Figure 5H), showed significant differences between the BDG-fed group and other control groups at 14 dpv. These results demonstrate that the oral administration of BDG activates potent adaptive immune signaling pathways via mucosal and systemic immune stimulation.

## 4 Discussion

Commercial FMD vaccines focus on inducing systemic immunity and do not induce mucosal immunity. This shortcoming makes it difficult to induce both mucosal and systemic immunity simultaneously. Synergistic effects must be achieved by simultaneously inducing mucosal and systemic immunity for a robust and long-lasting immune response in the host. As mucosal and systemic immunity interact to enhance the overall immunity of the host, an ideal vaccine should be able to simultaneously induce both mucosal and systemic immunity (36). Various vaccines and adjuvants (immunostimulants) were developed to induce mucosal immunity. Several studies showed that IM vaccination can induce robust mucosal immunity; however, the mucosal immunity is lower than that induced by direct mucosal stimulation via the mucosal route (6, 8, 37). Thus, we administered BDG orally in combination with vaccination to improve of the FMD vaccine and robustly induce systemic immunity, thereby eliciting a potent adaptive immunity.

We monitored changes in BW and food intake and calculated the FER in mice to evaluate the side effects of the oral administration program implemented in this study. No significant differences were observed between the BDG-fed group and the other control groups (Supplementary Tables 2 and 3). FER is commonly used to evaluate the *in vivo* toxicity of natural and synthetic products. The level of toxicity of the existing and new substances was defined by evaluating their *in vivo* toxicity (38, 39). Thus, the oral administration schedule of BDG in the BDG-fed group did not cause serious adverse effects in the host. The results of liver and kidney function tests conducted to evaluate adverse effects according to the oral administration schedule of BDG in target animals (pigs) showed that most values for all groups were within the normal range (Table 1) (40, 41). The pigs used in the experiment were Landrace pigs, and the normal range of AST in the serum of pigs is known to be 27.2–89.9 U/L. The AST level of the negative control group was found to be slightly higher than that of the vaccine-only and BDG-fed groups. This can be inferred to be due to the result of external factors, such as vaccination programs for other vaccines besides the FMD vaccine and stress, although it is considered not to be a critical problem because it was observed within the normal range. Therefore, oral administration of BDG to pigs does not induce harmful effects on the host.

In mice, we confirmed that the BDG-fed group showed a faster conversion to Ab-positivity for SP-specific Abs and virus-specific VN titers compared to the other control groups (Figure 1). Ab and VN titers are indicators of the success of a vaccination strategy. High Ab titers indicate a strong affinity between the B cells and the antigen of the host, signifying the induction of robust humoral immunity (42–44). VN titers also play a role in binding to the virus and blocking infection, and high VN titers suggest that the immune response induced by the vaccine can provide host defense against viral infection (45). Therefore, oral administration of BDG induces robust adaptive immunity by enhancing humoral immunity in mice. Since FMDV serotype O is the most frequently occurring FMDV serotype in South Korea, we used FMDV serotype O in the subsequent mouse challenge experiments (46). Although field FMDV is not susceptible to mice, FMDV strains that are infectious to mice were selected and cultured in previous experiments to perform mouse challenge experiments (47). In the these experiments, the BDG-fed group showed higher survival rates than vaccine-only group (Figure 2). Oral administration of BDG enhances the systemic immunity induced by vaccination, leading to robust long-term host defense, whereas vaccination alone induces ambiguous host defense. Consistent with previous vaccine challenge studies (48, 49), the BDG-fed group had higher survival rates and preserved body weight compared to the control groups in this study. High survival rates combined with weight preservation strongly indicate clinical protection (50, 51). Furthermore, the elevated Ab and VN titers at the time of challenge (84 dpv) support the high survival rate of the BDG-fed group.

Unlike SPF animals such as mice, farm pigs receive vaccines for other field viruses in addition to the FMD vaccine. So, farm pigs are vaccinated with a booster shot with the aim of eliciting a potent immune response specific to the antigen. The primary aim of vaccination is to establish immune memory against specific pathogens. T and B cells play crucial roles in adaptive immunity development (52). Memory B cells generate high-affinity VN Abs, effectively preventing pathogens from infecting cells. Memory T cells expand rapidly and use cytotoxic functions to eliminate pathogens, supporting infection prevention (53, 54). Consequently, BDG intake enhances FMD vaccine efficacy by stimulating both T and B cells, thereby promoting a stronger adaptive immune response (Figure 3). Oral administration of BDG significantly upregulated the production of SIgA in both mouse and pig sera. SIgA is widely known as a marker of mucosal immunity. SIgA is secreted primarily from Peyer’s patches and intestinal lymphoid tissues (55). FcαRI is the SIgA receptor and is expressed on a variety of cell types, including neutrophils and macrophages (56). SIgA eliminates pathogens by immune exclusion through agglutination, capture, and clearance processes or colonization (57, 58). Thus, the enhanced secretion of SIgA induced by oral administration of BDG may serve as the background for the higher Ab and VN titers observed in the BDG-fed group compared to those of the other control groups, as shown in Figures 1, 3, 4.

The gene expression levels of cytokines associated with mucosal immunity elicited by the oral administration of BDG were evaluated at 14 dpv using qRT-PCR. The background for collecting samples at 14 dpv was that cellular and adaptive immune indices (Ab and VN titers) showed significant differences at 14 dpv between the BDG-fed group and other control groups evaluated in pigs. Therefore, we speculated that gene expression levels would show significant differences. Among the evaluated cytokines, IL-2, IL-4, IL-18, and IFNγ primarily contribute to cellular and humoral immunity. These cytokines promote the maturation and differentiation of adaptive immune cells (T cells and memory cells) and induce Ab-mediated immune responses (59–62). IL-12 is divided into two subunits, IL-12p35 and IL-12p40. IL-12p40 assembles with IL-23p19 to produce IL-23 (63, 64). IL-23 exerts significant effects on the host immune system by inducing Th17 cells, which secrete and express IL-17A and IL-23R (65). In addition to Th17 cells, various innate immune cells, including γδ T cells, natural killer T cells, and innate lymphoid cells (ILCs), recognize IL-23 and respond to pathogen invasion (66, 67). The IL-17 family is mainly secreted by lymphocytes and serves as a link between the immune system and the mucosa. IL-17A promotes mucosal immunity by activating the IL-17A receptors expressed on mesenchymal and epithelial cells (68).

Various adjuvants were studied in human vaccines, including immunostimulants such as pattern-recognition receptor (PRR) ligands, cytokines, and small molecules, as well as antigen delivery systems such as lipid nanoparticles, polymeric particles, protein nanocages, and inorganic nanocarriers (69, 70). In veterinary vaccines (particularly FMD vaccines), the adjuvants currently in use include oil-based emulsions, saponin, and aluminum gel. Several PRR ligands and cytokines are being studied as potential adjuvants for FMD vaccines. FMD vaccines contain substantial amounts of oil-based emulsion, which can cause issues such as injection-site necrosis and accelerated antigen degradation. In previous studies, we included BDG in the FMD vaccine formulation while reducing the dosage of other adjuvants to enhance vaccine efficacy while minimizing adverse effects (13).

In this study, we further assessed the efficacy of BDG administered orally as part of efforts to develop an oral FMD vaccine. We demonstrated that the BDG intake induced mucosal immunity enhanced the systemic immunity induced by FMD vaccination, resulting in robust adaptive (cellular and humoral) immunity. However, this study has several limitations. Frist, the FMD vaccine was administered intramuscular rather than orally. To administer an FMD vaccine orally, the vaccine must easily pass through the mucosal barrier and withstand harsh environments such as stomach acid. Therefore, future studies should develop a new vaccine delivery system to overcome these limitations, and manufacture and evaluate its efficacy as an oral vaccine. Second, this study used only mice and pigs as animal models, and the vaccine was limited to the FMD vaccine combined with oral BDG. Thus, potential challenges exist in extrapolating the current findings to those of other species or vaccines. Further studies in different livestock species and vaccines are needed to prevent and control a wide range of diseases using oral BDG. Overall, this study provides a new direction for the development of next-generation vaccine strategies, including oral vaccines.

## Data Availability

The original contributions presented in the study are included in the article/Supplementary material; further inquiries can be directed to the corresponding author.
