# Supplementary material for: Oral β-D-glucan potentiates systemic immune responses to intramuscular foot-and-mouth disease vaccination
Source: Front Vet Sci. 2026 Jan 8;12:1701909. doi: 10.3389/fvets.2025.1701909 (PMC12825052; doi:10.3389/fvets.2025.1701909)
Supplement: Supplementary file 1 [file Data_Sheet_1.PDF]

Supplementary Material

Oral β-D-glucan potentiates systemic immune responses to intramuscular foot-and-mouth disease vaccination

Hyeong Won Kim[1], So Hui Park[1], Mi-Kyeong Ko[1], Seokwon Shin[1], Jong-Hyeon Park[1], Min Ja Lee[1*]

[1]Center for Foot-and-Mouth Disease Vaccine Research, Animal and Plant Quarantine Agency, 177 Hyeoksin 8-ro, Gimcheon-si, Gyeongsangbuk-do 39660, Republic of Korea

*** Correspondence:** Min Ja Lee: herb12@korea.kr

1 Supplementary Tables

1.1 Supplementary Table

Supplementary Table 1. List of primer sequences for qRT-PCR.

Target	Forward/Reverse	Sequence (5′-3′)	Length (mer)
IL-2	IL-2 F	AAGCTCTGGAGGGAGTGCTA	20
	IL-2 R	CAACAGCAGTTACTGTCTCATCA	23
IL-4	IL-4 F	CTCACCTCCCAACTGATCCC	20
	IL-4 R	TGTGTCCGTGGACGAAGTTG	20
IL-12p40	IL-12p40 F	GGAGTATAAGAAGTACAGAGTGG	23
	IL-12p40 R	GATGTCCCTGATGAAGAAGC	20
IL-17A	IL-17A F	CTCGTGAAGGCGGGAATCAT	20
	IL-17A R	GGTGTGCTCCGGTTCAAGAT	20
IL-18	IL-18 F	AGCTGAAAACGATGAAGACCTG	22
	IL-18 R	AAACACGGCTTGATGTCCCT	20
IL-23p19	IL-23p19 F	CCATATCCAGTGCGGGGATG	20
	IL-23p19 R	AGGCCTTGGTGGATCCTTTG	20
IL-23R	IL-23R F	TCCCTCATTGCAAAGCACAA	20
	IL-23R R	GCATCTCCTCTTGCAAGCAAAT	22
IFNγ	IFN-γ F	GCCATTCAAAGGAGCATGGAT	21
	IFN-γ R	CTGATGGCTTTGCGCTGGAT	20
HPRT	HPRT F	CCCAGCGTCGTGATTAGTGA	20
	HPRT R	GCCGTTCAGTCCTGTCCATA	20

Supplementary Table 2. Body weight gain of mice treated with or without β-D-glucan (BDG) by oral administration for 56 days post vaccination (dpv).

Group	0 dpv	7 dpv	14 dpv	21 dpv	28 dpv	56 dpv
NC	18.68±0.56	19.93±0.56	20.85±0.85	21.39±0.86	21.54±0.62	23.13±1.19
PC	19.10±0.68	20.84±0.68	21.00±0.98	21.57±0.78	21.70±0.79	24.04±0.62
Exp	18.63±0.47	20.13±0.42	20.59±0.73	21.19±0.81	21.24±0.58	21.36±0.41

C57BL/6 mice (females, 6–7 weeks old, *n* = 5/group) were divided into three groups, namely, a negative control (NC) group, a positive control (PC) group, and an experimental (Exp) group. Body weights were measured once a week from 0 dpv to 28 dpv and then at 56 dpv at the same time and conditions. Data are represented as the mean ± SEM of triplicate measurements (*n* = 5/group). Statistical analyses were performed using two-way ANOVA, followed by Tukey's *post-hoc* test.

dpv, days post vaccination; NC, negative control; PC, positive control; Exp, experimental

Supplementary Table 3. Weight gain, food intake, and food efficiency ratio (FER) of mice treated with β-D-glucan (BDG) by oral administration for 56 days post vaccination.

Group	Weight gain (g/56 dpv)	Food intake (g/56 dpv)	FER
NC	4.45±1.37	121.57±3.45	3.66±1.12
PC	4.85±1.15	152.86±2.68	3.17±0.75
Exp	4.63±0.70	125.93±2.25	3.68±0.43

C57BL/6 mice (females, 6–7 weeks old, *n* = 5/group) were divided into three groups, namely, a negative control (NC) group, a positive control (PC) group, and an experimental (Exp) group. Body weight a 3.45d feed intake were measured once a week from 0 dpv to 28 dpv and then at 56 dpv at the same time and conditions. 1) FER, food efficiency ratio {FER=Body weight gain (g/dpv) / food intake (g/dpv) *100}. Data are represented as the mean ± SEM of triplicate measurements (*n* = 5/group). Statistical analyses were performed using two-way ANOVA, followed by Tukey's *post-hoc* test.

dpv, days post vaccination; FER, food efficiency ratio; NC, negative control; PC, positive control; Exp, experimental

Supplementary Table 4. Experimental strategies employing murine and porcine models

Mouse (C57BL/6N, 6–7 weeks old, ♀, *n* = 5/group)

Group	Vaccination (IM)	Daily administration (Oral)	Weekly administration (Oral)	Bleeding	Viral challenge (IP)	Monitoring
**NC**	PBS (0 dpv)	Water (0–28 dpv)	Water (35, 42, 49, 56 dpv)	0, 7, 14, 21, 28, 35, 42, 56, 70, 84 dpv	O/VET/2013 (84 dpv/0 dpc)	0–84 dpv, post-challenge
**PC**	FMD vaccine (0 dpv)	Water (0–28 dpv)	Water (35, 42, 49, 56 dpv)	0, 7, 14, 21, 28, 35, 42, 56, 70, 84 dpv	O/VET/2013 (84 dpv/0 dpc)	0–84 dpv, post-challenge
**Exp**	FMD vaccine (0 dpv)	BDG (0–28 dpv)	BDG (35, 42, 49, 56 dpv)	0, 7, 14, 21, 28, 35, 42, 56, 70, 84 dpv	O/VET/2013 (84 dpv/0 dpc)	0–84 dpv, post-challenge

Pig (Landrace pig, 8–9 weeks old, *n* = 4−5/group)

Group	Vaccination	Daily oral administration	Weekly oral administration	Bleeding	Viral challenge	Monitoring
**NC**	PBS (0 dpv)	Water (0–28 dpv)	Water (35, 42, 49, 56 dpv)	0, 7, 14, 21, 28, 35, 42, 56, 70, 84 dpv	– (Not performed)	0–84 dpv
**PC**	FMD vaccine (0 dpv)	Water (0–28 dpv)	Water (35, 42, 49, 56 dpv)	0, 7, 14, 21, 28, 35, 42, 56, 70, 84 dpv	– (Not performed)	0–84 dpv
**Exp**	FMD vaccine (0 dpv)	BDG (0–28 dpv)	BDG (35, 42, 49, 56 dpv)	0, 7, 14, 21, 28, 35, 42, 56, 70, 84 dpv	– (Not performed)	0–84 dpv

dpv, days post vaccination; NC, negative control; PC, positive control; Exp, experimental

2 Supplementary Figures

2.1 Supplementary Figure

Supplementary Figure 1. BDG intake enhances the efficacy of the FMD vaccine, eliciting high secretory IgA concentrations in the saliva of mice.

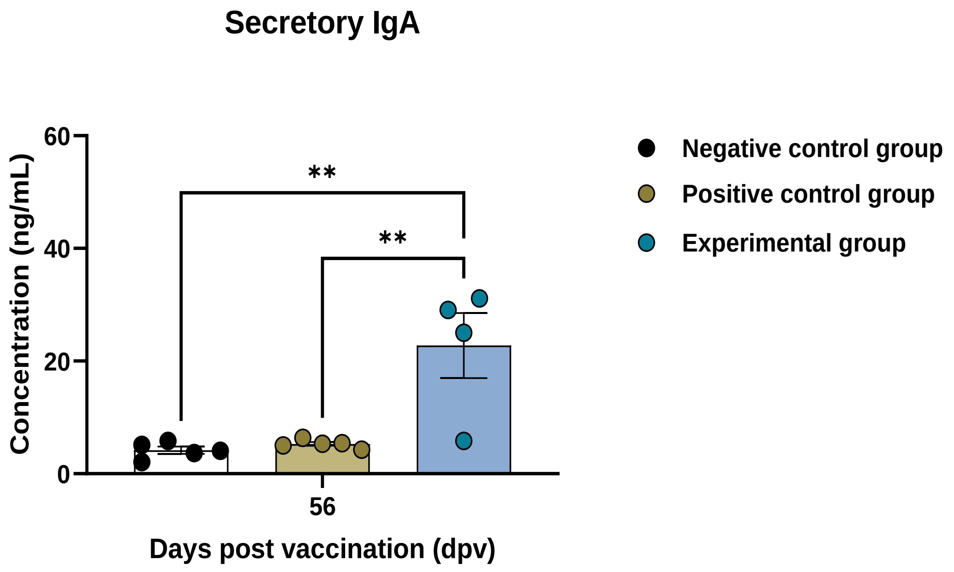

The experimental strategy and methods are described in the legends of Figures 1A and 3A. Data are presented as the mean ± SEM of triplicate measurements (n = 4–5/group). Statistical analyses were performed using two-way ANOVA, followed by Tukey's *post-hoc* test. $^{**}p <$ 0.01.
